# Replacing Helper Lipids With Cationic Lipids Enhances mRNA Lipid Nanoparticles Stability in Solution

**DOI:** 10.1002/advs.202511637

**Published:** 2025-10-30

**Authors:** Rui Chen, Letao Xu, Nan Zhang, Haitao Yu, Xing Wang, Jiali Zhai, Fiona Whelan, Phillip Elliott, Chun‐Xia Zhao

**Affiliations:** ^1^ School of Chemical Engineering The University of Adelaide Adelaide South Australia 5000 Australia; ^2^ Adelaide Microscopy, School of Biological Sciences The University of Adelaide Adelaide South Australia 5000 Australia; ^3^ STEM College, RMIT University Melbourne Victoria 3000 Australia; ^4^ BioCina Pty Ltd Adelaide South Australia 5031 Australia

**Keywords:** cryogenic electron microscopy, lipid nanoparticles, microstructure, mRNA, stability study

## Abstract

Messenger RNA lipid nanoparticles (mRNA‐LNPs) have achieved remarkable success in clinical vaccination efforts to curb the COVID‐19 pandemic, and have attracted tremendous interest from both industry and academia to broaden their biomedical applications. However, their storage and transportation rely heavily on the cold chain to prevent rapid degradation in non‐frozen solutions, posing significant challenges for logistics and accessibility. Therefore, enhancing the in‐solution stability of mRNA LNPs is crucial. Herein, the role of helper phospholipids is investigated in storage stability of mRNA LNPs. By ratiometrically replacing the helper phospholipids (DSPC) with other alternative lipids (DOPS, DOTAP, and DOPE) in commercially available mRNA LNP formulation, enhanced in‐solution stability was observed with cationic lipid (DOTAP) substitution at 42 °C, room temperature (22 °C), and 4 °C. Further correlation of stability with microstructure analysis using cryogenic electron microscopy revealed that partial replacement with DOTAP enhances structural stability by promoting the formation of intact LNPs and reducing the occurrence of “bleb”‐like structures. Overall, the study provides insights into the structure‐stability relationship in mRNA LNPs, and offers new strategies to address the storage limitations of mRNA‐LNP products in the future.

## Graphical abstract

Partial Replacement of phospholipid lipid by cationic lipid improves storage stability of commercial mRNA LNPs by stabilizing their internal microstructure.

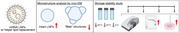

## 1 Introduction

Lipid nanoparticles (LNPs) have recently emerged as the most clinically advanced nanocarriers for messenger RNA (mRNA) delivery, mainly due to the approval and successful deployment of mRNA‐LNP vaccines during the COVID‐19 pandemic.^[^ ^1^, ^2^, ^3^, ^4^ ^]^ Encouraged by their clinical success, mRNA LNPs have been actively explored in broader biomedical applications such as cancer therapy.^[^ ^5^, ^6^, ^7^ ^]^ While LNPs are designed to protect and deliver mRNA, stability remains a persistent issue, as mRNA‐LNP products lose activity quickly in solution when stored at room temperature (RT).^[^ ^8^ ^]^ Therefore, the two clinically approved mRNA‐LNP COVID vaccines: BNT162b2 (Pfizer‐BioNTech) and mRNA‐1273 (Moderna), require cold‐chain logistics for storage and transport, resulting in high costs and restricted global accessibility.^[^ ^9^ ^]^

To address the need of stable mRNA‐LNPs under non‐frozen storage conditions, lyophilization offers a potential solution by eliminating water from mRNA‐LNPs, thereby preventing mRNA hydrolysis and extending shelf life.^[^ ^10^, ^11^, ^12^ ^]^ Recent studies have shown that lyophilized mRNA‐LNP vaccines can retain their physicochemical properties and bioactivities after storage at RT for 3 to 6 months.^[^ ^13^, ^14^ ^]^ Nevertheless, lyophilization is costly and time‐consuming, requiring temperature control during the multiple drying processes.^[^ ^15^ ^]^ Additional concerns, such as a change in particle size and efficacy upon reconstitution of lyophilized mRNA‐LNPs, also need to be addressed. Other efforts have been directed toward investigating the components of LNPs responsible for mRNA stability.^[^ ^16^, ^17^, ^18^ ^]^ Packer et al. identified aldehyde impurities arising from the oxidation of typical tertiary amines in ionizable lipids, which inhibit mRNA translation.^[^ ^19^ ^]^ The use of piperidine‐based ionizable lipids to reduce the generation of aldehyde impurities has been shown to improve storage stability of mRNA‐LNPs in solution.^[^ ^20^ ^]^

Helper phospholipids, which stabilize LNP structures, have been modified to alter tissue distribution and cellular uptake tropism of LNPs.^[^ ^21^, ^22^ ^]^ For instance, selective organ targeting (SORT) LNPs rely on the chemical structure of additional charged lipids or phospholipids to achieve targeted organ delivery.^[^ ^23^, ^24^, ^25^ ^]^ Moreover, either increasing the proportion of the traditional helper phospholipid 1,2‐distearoyl‐*sn*‐glycero‐3‐phosphocholine (DSPC) or replacing it with sphingomyelin has been shown to produce a unique bilayer microstructure that enhanced LNP stability in serum.^[^ ^26^ ^]^ Based on these findings, we hypothesized that optimizing helper lipids can influence the complexing of mRNA and lipids within mRNA‐LNPs and potentially improve their storage stability in solution at non‐frozen conditions.

In this study, we replaced partial and entire DSPC in the two commercialized mRNA‐LNP formulations with other alternative lipids with negative (DOPS), neutral (DOPE) and positive charges (DOTAP), respectively. The LNP formulations were incubated at elevated temperature (42 °C), RT (22 °C), and refrigeration (4 °C), and were assessed through a combination of particle size, encapsulation efficiency, mRNA integrity, and cell transfection assay. We observed significant enhancement in mRNA‐LNP stability for the formulation with 50% of the DSPC replaced by the positively charged 1,2‐dioleoyl‐3‐trimethylammonium propane (DOTAP) at 42 °C, as well as improvement in stability at 22 and 4 °C compared to the counterpart commercial formulation. Further mechanistic studies revealed that partial replacement of DSPC by DOTAP inhibited the formation of “bleb” structures for both freshly prepared mRNA LNPs and those subjected to stability testing. Our results demonstrated a facile and effective approach to enhancing mRNA‐LNP stability by replacing helper lipids, offering a new strategy of optimizing mRNA‐LNP formulations with extended storage stability for practical applications.

## 2 Results

**Figure 1:**
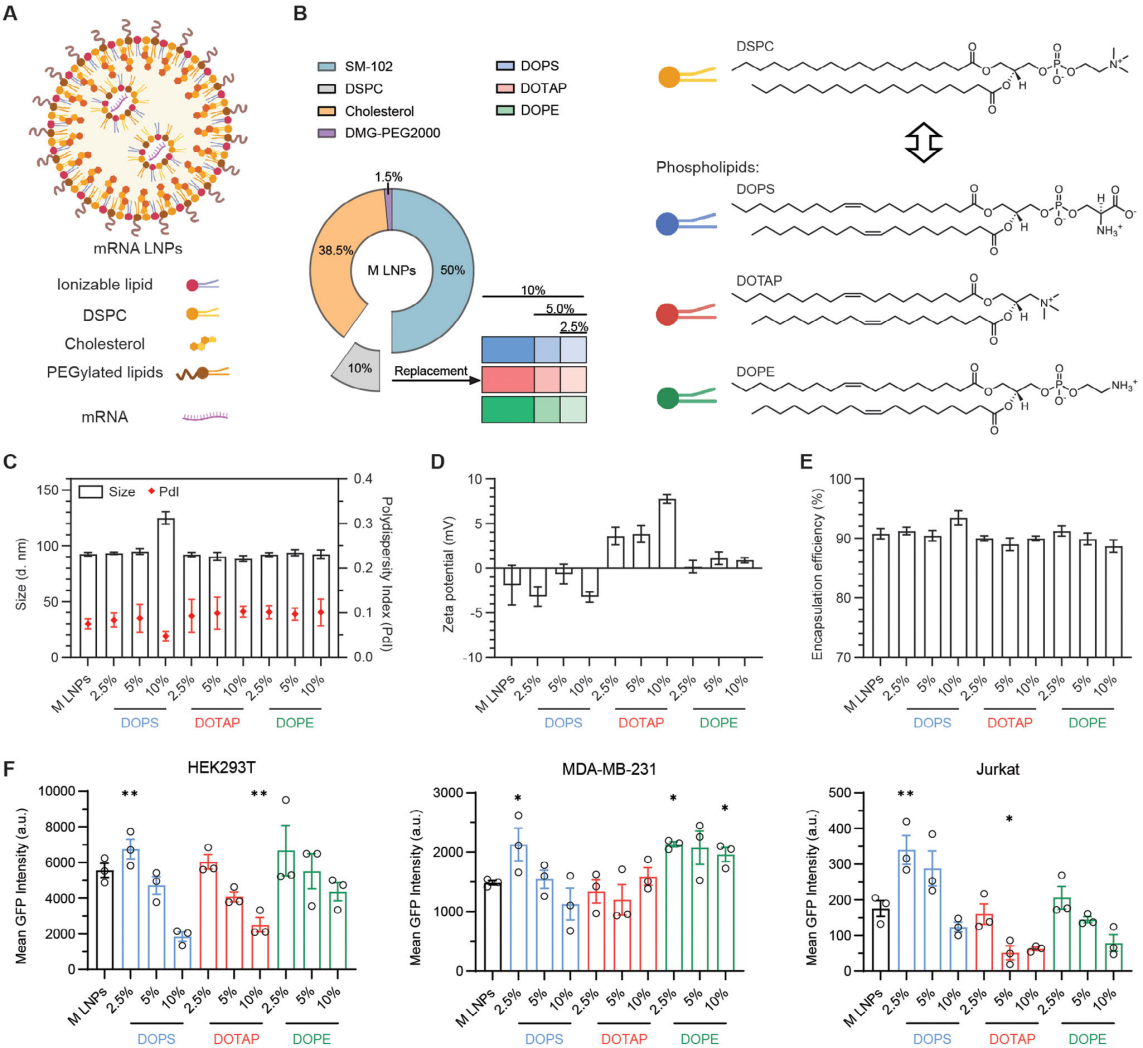
Design and evaluation of mRNA‐LNP formulations with DSPC replaced by various alternative lipids: DOPS, DOTAP, and DOPE. A) Schematics of lipid components of Moderna LNPs (M LNPs). B) Formulation of M LNPs (DSPC) and M LNPs with the replacement of alternative lipids: DOPS, DOTAP, and DOPE. C) Particle size, D) zeta potential, and E) encapsulation efficiency of the mRNA LNPs. (F) Mean fluorescence intensity of green fluorescent protein (GFP) in HEK293T, MDA‐MB‐231, and Jurkat cells at 24 h post‐transfection of the mRNA‐LNPs. Three batches of different LNPs formulations were prepared and tested independently (^*^ *p *<0.05, ^**^ *p* <0.01).

LNPs carrying mRNA typically involve the self‐assembly of mRNA with four lipid components, including ionizable lipids, DSPC, cholesterol, and PEGylated lipids (**Figure** **1**A). We first conducted some preliminary studies by incubating mRNA with each of the four lipid components individually and evaluated their effects on mRNA degradation. Surprisingly, we observed the most pronounced mRNA degradation upon exposure to DSPC in PBS, whereas the commercial‐grade mRNA alone remained stable (Figure S1, Supporting Information). To better understand the role of the helper phospholipid DSPC in mRNA‐LNP degradation, we replaced DSPC with various charged and neutral alternative lipids and compared their storage stability. We selected three types of alternative lipids DOPS, DOTAP, and DOPE, with different charges,^[^ ^27^ ^]^ to replace 25%, 50% and 100% of the DSPC (corresponding to 2.5%, 5% and 10% of total lipids) in the Moderna formulation mRNA‐1273 (M LNPs, Figure 1B). Their hydrodynamic particle size, polydispersity index (PdI), zeta potential, and encapsulation efficiency of the as‐formulated mRNA LNPs were characterized and compared them with benchmark Moderna formulation M LNPs (Figure 1C–E). It is noted that 10% DOPS resulted in a size increase as compared to other LNPs, and DOTAP replacement at different proportions induced the formation of LNPs with a slightly positive charge.

Next, we used mRNA encoding enhanced green fluorescent protein (mRNA‐eGFP) as a reporter to examine in vitro transfection of the modified LNP formulations on HEK293T (epithelial embryonic kidney), MDA‐MB‐231 (epithelial breast cancer), and Jurkat (T lymphoblast) cells by flow cytometry analysis. As shown in Figure 1F and Figures S2–S4 (Supporting Information), replacement of helper lipids with alternative lipids had varying impacts on mRNA transfection, depending on the cell types. Particularly, replacing DSPC with DOPE or 2.5% DOPS can enhance mRNA transfection in MDA‐MB‐231 and Jurkat cells, respectively. However, complete replacement of DSPC by DOPS, DOTAP, or DOPE substantially decreased GFP expression in all the three types of cells.

**Figure 2:**
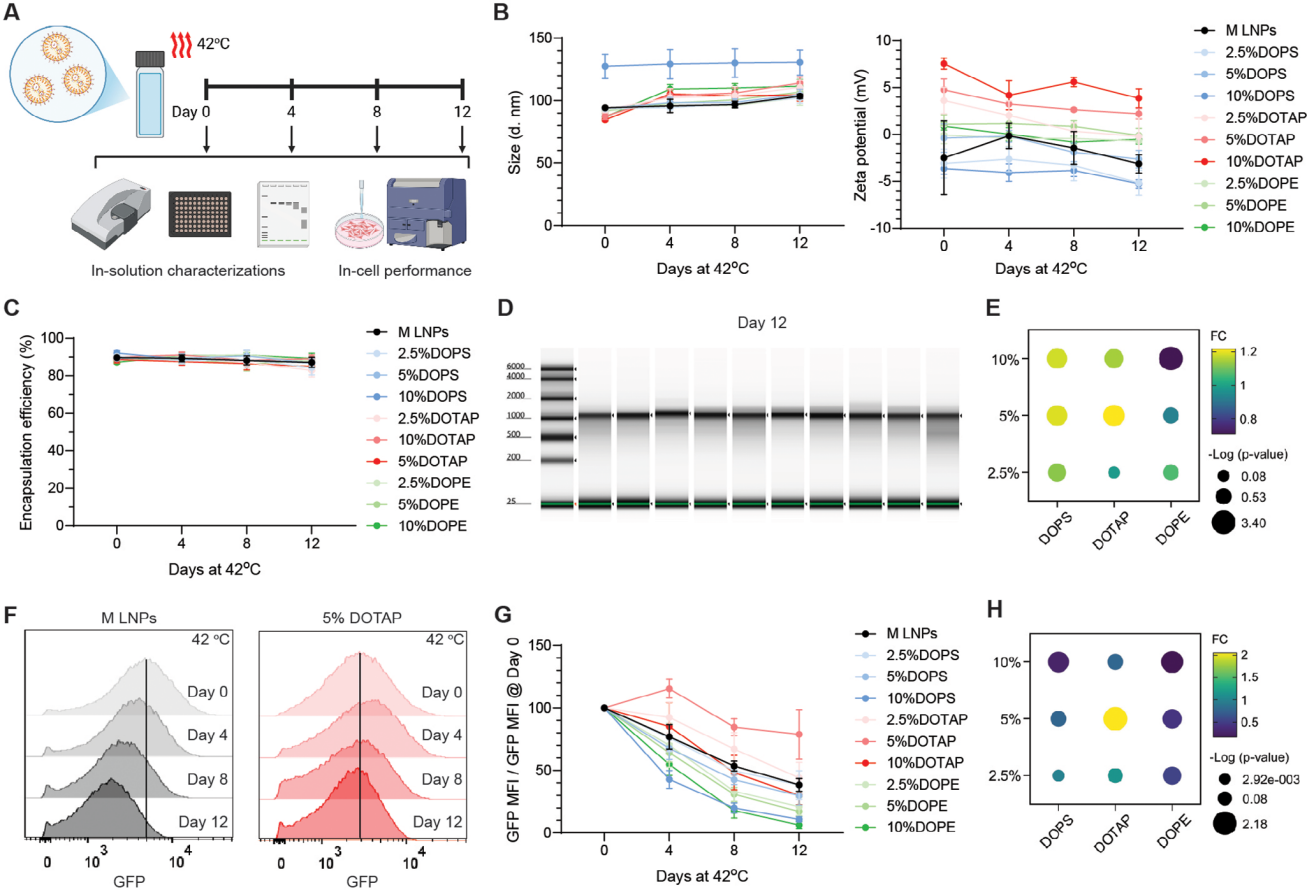
Stability of M LNPs and the LNPs with DSPC replacement at 42 °C, i.e., elevated temperature condition. A) Schematic illustration of sample collections and tests including DLS, RiboGreen assay, RNA electrophoresis and cell transfection followed by flow cytometry analysis. B) Hydrodynamic particle sizes and zeta potentials of the LNPs upon exposure to 42 °C for 0, 4, 8 and 12 days. C) Encapsulation efficiencies of the LNPs during the stability test. D) RNA electrophoresis of the encapsulated mRNAs in the LNPs on day 12 (bands from left to right: M LNPs, M LNPs with 2.5%/5%/10% DOPS, M LNPs with 2.5%/5%/10% DOTAP, and M LNPs with 2.5%/5%/10% DOPE). E) Fold change (FC) and –log(p‐value) of intact mRNA for the phospholipid‐replacement formulations in comparison to commercial M LNP formulation (i.e., 10% DSPC) on day 12. F) Concatenated histograms of GFP signal for HEK293T cells treated with M LNPs and their 5% DOTAP replacement. G) Normalized mean fluorescence intensity (MFI) of GFP signal in HEK293T cells treated with the collected LNPs. (H) FC and –log(p‐value) value of in‐cell performance for the phospholipid replacement formulations in comparison to standard M LNP formulation (i.e., 10% DSPC) on day 12. Three batches of LNPs were prepared and tested independently.

To study the storage stability of mRNA LNPs, we first incubated various LNP solutions at an elevated temperature of 42 °C to accelerate the degradation process. Samples of M LNPs as well as all the DOPS, DOTAP, and DOPE replacement LNPs were collected on day 0, 4, 8, and 12, and characterized using several in‐solution and in‐cell assays to evaluate their stability (**Figure** **2**A). Only minimal changes were observed in hydrodynamic sizes, zeta potentials (Figure 2B) and encapsulation efficiencies (Figure 2C) among all formulations of mRNA LNPs. On day 12, electrophoresis analysis of the mRNA extracted from mRNA LNPs showed some smear bands below 1000 nt, corresponding to fragments of degraded mRNAs (Figure 2D). We quantified the percentages of the intact mRNA based on grey scale intensities of main peak and smear bands of different LNP formulations, then calculated the fold change (FC) as compared to the commercial M LNPs (Figure 2E). Statistical significance was also calculated and represented by ‐log(p‐value). It must be noted that the encapsulated mRNA in LNPs with 5% DOTAP replacement retained 68.1% intact mRNA on day 12, which is 21% higher than that of the M LNPs with a ‐log(p‐value) of 2.25 (*p* = 0.0056).

Furthermore, we transfected HEK293T cells with the collected mRNA‐eGFP LNPs and evaluated GFP signal change of the cells to represent in‐cell functional stability. Concatenated histograms of GFP in HEK293T cells represented a slight decrease for M LNPs with 5% DOTAP (Figure 2F). To eliminate the influence of differences in transfection efficacy caused by phospholipid replacement, as shown in Figure 1F, we normalized the mean GFP intensity at different time points to that of day 0 (Figure 2G). Statistical analysis of fold change and significance was also performed by comparing different formulations with M LNPs (Figure 2H). M LNPs with 5% DOTAP showed a 105% enhancement with a ‐log(p‐value) of 2.18 (*p* = 0.0066). In summary, consistent trend of change has been found in RNA electrophoresis and in‐cell functional stability, which collectively demonstrated that degradation of the encapsulated mRNA was one of the main reasons of instability, and 5% DOTAP replacement improved the storage stability of M LNPs under elevated temperature conditions.

**Figure 3:**
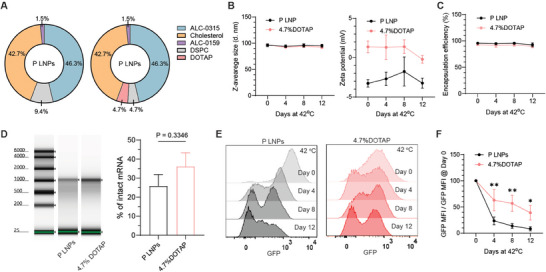
Stability study of Pfizer‐BioNTech LNPs (P LNPs) and the LNPs with 4.7% DOTAP at 42 °C. A) Schematic illustration of the P LNP formulation. B) Hydrodynamic particle sizes and zeta potentials of the LNPs after being exposed to 42 °C for 0, 4, 8, and 12 days. C) Encapsulation efficiencies of the LNPs during the stability test. D) RNA electrophoresis and quantitative analysis of the encapsulated mRNAs on day 12. E) Concatenated histograms of GFP signal for HEK293T cells treated with P LNPs and P LNPs with 4.7% DOTAP. G) Normalized MFI of GFP signal in HEK293T cells treated with the collected LNPs. Three batches of LNPs were prepared and tested independently (^*^ *p* <0.05, ^**^ *p* <0.01).

We next investigated the applicability of DOTAP replacement to improve the storage stability of another commercially available mRNA‐LNP system BNT162b2 (Pfizer‐BioNTech). DOTAP was used to replace half of the DSPC in Pfizer‐BioNTech mRNA LNPs (P LNPs), resulting in a formulation of P LNPs with 4.7% DOTAP (**Figure** **3**A). After incubating the original P LNPs and those with 4.7% DOTAP at 42 °C, we didn't observe any significant changes in hydrodynamic sizes, zeta potentials, and encapsulation efficiencies (Figure 3B,C). Notably, RNA electrophoresis of the LNP samples on day 12 showed a trend of increase in percentages of intact mRNA for the LNPs with 4.7% DOTAP. Moreover, GFP signals of HEK293T cells treated with P LNPs and the LNPs with 4.7% DOTAP at different time points were characterized and compared (Figure 3E). Normalized results indicated that a significantly delayed degradation of in‐cell function was achieved by P LNPs with 4.7% DOTAP. In addition, we assessed cell viability of the HEK293T cells treated with M LNPs, P LNPs, and their DOTAP replacement formulations (Figure S5, Supporting Information). Over 80% cell viability was observed in all four formulations at various concentrations, and addition of DOTAP didn't increase cytotoxicity due to their low percentages in total lipids of LNPs.

**Figure 4:**
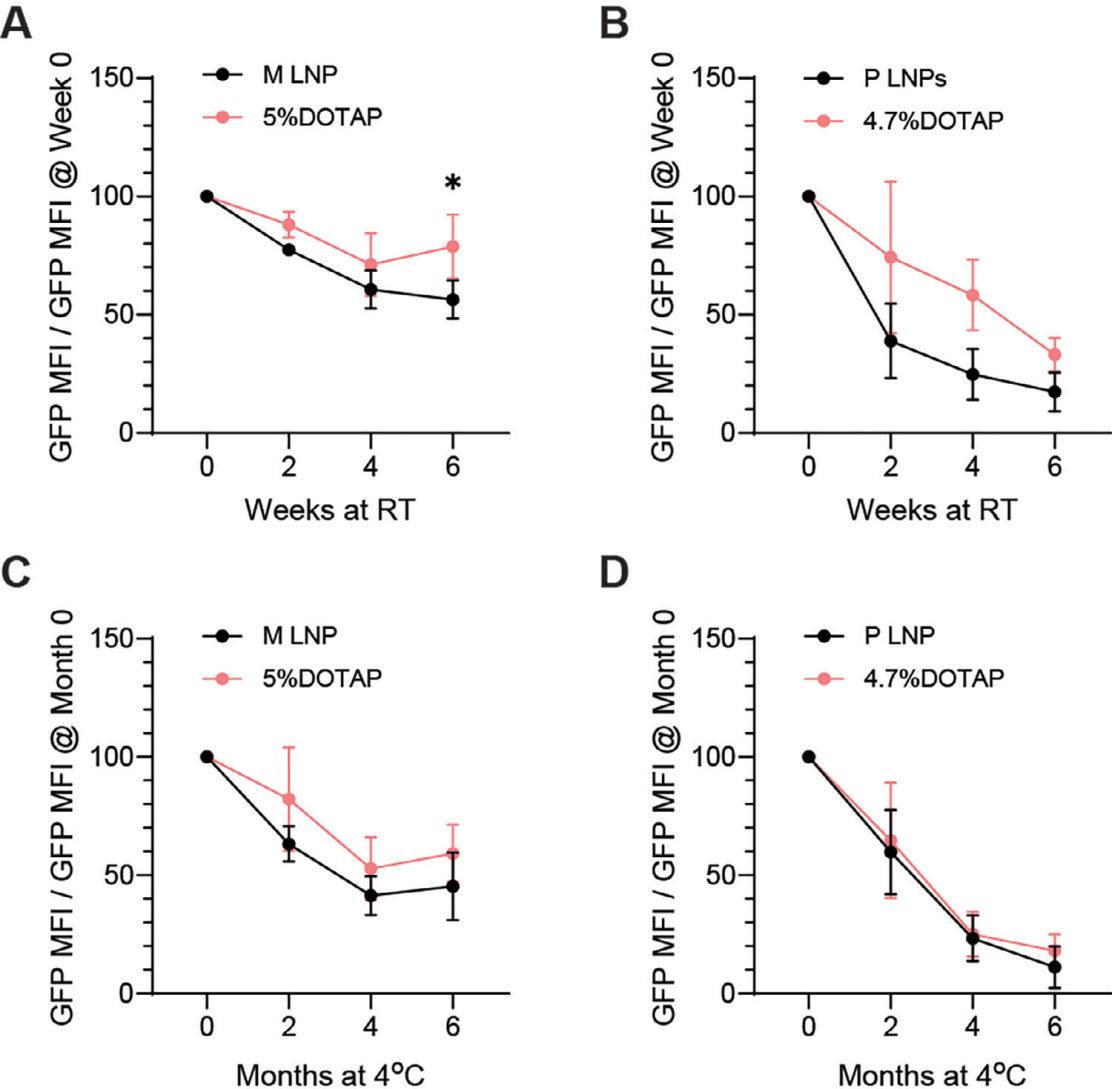
Stability study of M LNPs and P LNPs with DOTAP replacement under RT and 4 °C. Normalized GFP signal of HEK293T cells treated with A) M LNPs and 5% DOTAP, B) P LNPs and 4.7% DOTAP collected from stability study at RT. Normalized GFP signal of HEK293T cells treated with C) M LNPs and M LNPs with 5% DOTAP, (D) P LNPs and PLNPs with 4.7% DOTAP collected from stability study at 4 °C. Three batches of different LNP formulations were independently prepared and tested per temperature condition (^*^ *p* <0.05).

Further stability studies of mRNA‐LNPs with DOTAP replacement were carried out under RT (22 °C) and 4 °C for up to 6 weeks and 6 months, respectively. Hydrodynamic size, zeta potential, and encapsulation efficiency of M LNPs, M LNPs with 5% DOTAP, P LNPs, and P LNPs with 4.7% DOTAP remained stable over the testing periods (Figure S6, Supporting Information). In terms of RNA electrophoresis, we were unable to detect any differences in the percentages of intact mRNA between standard formulations and those with DOTAP replacement. When comparing the storage stability under RT by checking their in‐cell performance, M LNPs with 5% DOTAP and P LNPs with 4.7% DOTAP only showed a trend of improvement as compared to M LNPs and P LNPs (**Figure** **4**A,B; Figures S7 and S8, Supporting Information). At 4 °C, there was a slight improvement of storage stability for M LNPs with 5% DOTAP, while replacement with DOTAP did not change stability of P LNPs for GFP translation in cells (Figure 4C,D; Figures S9 and S10, Supporting Information).

**Figure 5:**
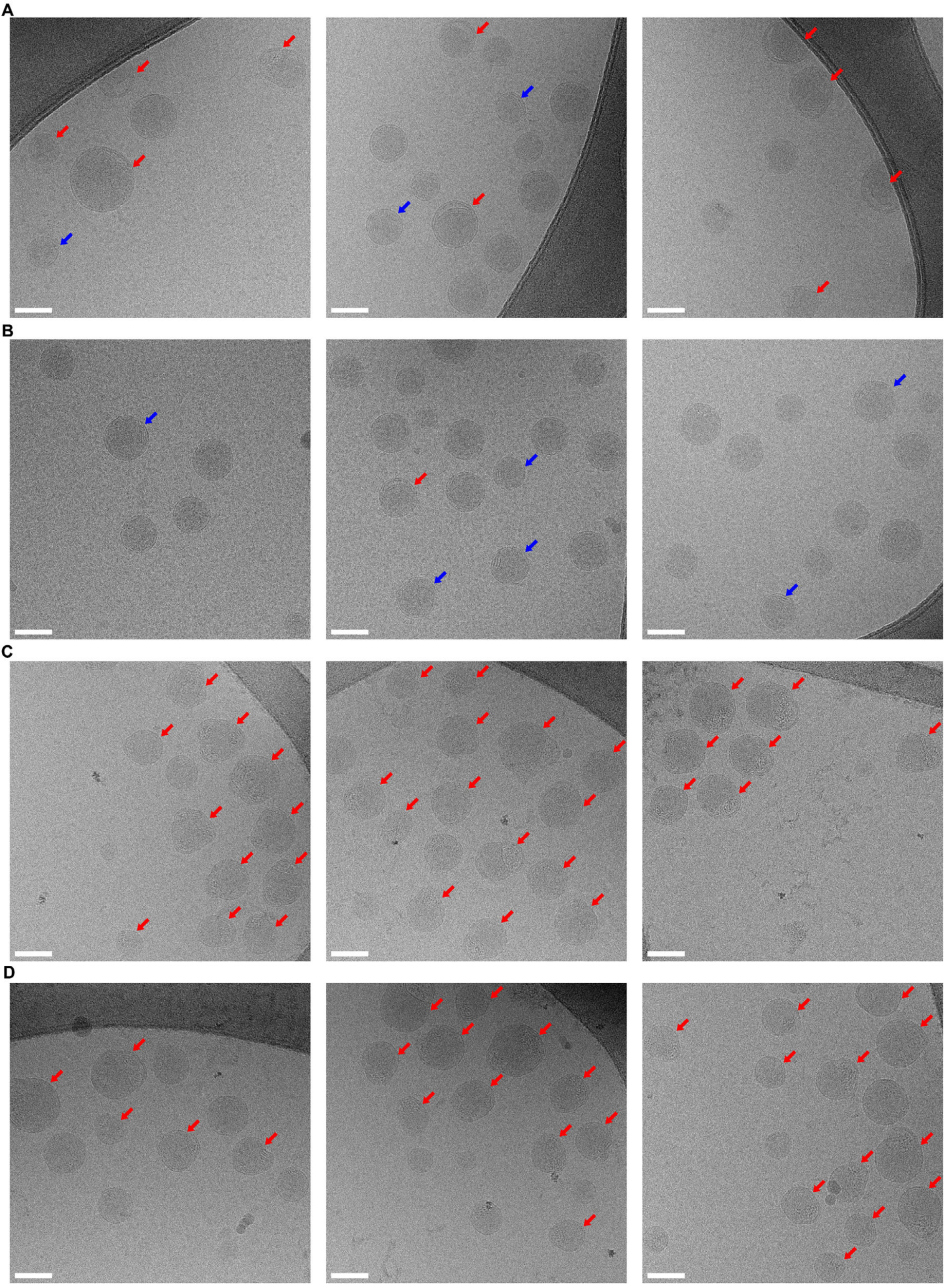
Microstructures of LNPs characterized by cryo‐EM. Representative images of freshly prepared A) M LNPs and B) M LNPs with 5% DOTAP; C) M LNPs, (D) M LNPs with 5% DOTAP after incubation at 42 °C for 4 days. Scale bar = 50 nm. Blue arrows indicate multi‐layer structure, red arrows indicate “bleb”‐like structure, intact LNPs are not labeled.

Given that we observed differences in surface charge and storage stability between M LNPs and M LNPs with 5% DOTAP, we furthered our investigations by comparing the microstructures of these two LNPs to explore the underlying mechanism. First, we used cryogenic electron microscopy (cryo‐EM), which has been frequently employed in the previous studies,^[^ ^28^ ^]^ to image M LNPs and M LNPs with 5% DOTAP dispersed in PBS. As shown in the representative images, M LNPs and M LNPs with 5% DOTAP represented an average particle size of 50–90 nm, which is relevant to their average sizes characterized by dynamic light scattering (**Figure** **5**A,B). By reviewing the images, we identified three types of microstructures in the mRNA‐LNP samples: 1) intact spheres, 2) multiple layers, and 3) “bleb” structures (Figure S11, Supporting Information). It is hypothesized that the multi‐layer structure is an intermediate between intact and “bleb”‐like LNPs. In addition, we obtained cryo‐EM images of those mRNA‐LNPs after incubation at 42 °C for 4 days (Figure 5C,D).

To perform statistical analysis and exclude bias of the imaging process, we analyzed over 300 LNPs at randomly picked grids from two preparations, and classified those nanoparticles into these three categories of structures. Fractions of different structures in M LNPs, M LNP with 5% DOTAP freshly prepared and after 42 ^°^C incubation were summarized in **Table** 1. Freshly prepared M LNPs are constituted by 53.2% intact LNPs, 11.5% LNPs with multi‐layer regions, and 35.3% “bleb”‐like LNPs.^[^ ^29^ ^]^ It must be noted that replacing DSPC with DOTAP at 5% of total lipids increased “intact spheres” by 18% and reduced “bleb” structures by 24.1%. We also measured and compared spacing of two adjacent layers in the multi‐layer structures of M LNPs and M LNPs with 5% DOTAP, which represents 3.96 and 3.86 nm without any significant difference (Figure S12, Supporting Information).

**Table 1:** Statistical analysis of cryo‐EM images of M LNPs and M LNPs with 5% DOTAP before and after incubation at 42 °C.

	Intact sphere	Multiple layers	"Bleb" structure
*Freshly prepared LNPs*:			
M LNPs	53.2%	11.5%	35.3%
M LNPs with 5% DOTAP	71.2%	17.6%	11.2%
*LNPs after incubation at 42 °C for 4 days*:			
M LNPs	13.7%	0.00%	86.3%
M LNPs with 5% DOTAP	28.1%	0.3%	71.6%

Synchrotron small‐angle X‐ray scattering (SAXS) was also utilized to characterize the internal structures of LNPs and mRNA LNPs.^[^ ^30^, ^31^, ^32^ ^]^ SAXS analysis of the M LNPs in PBS buffer in Figure S13 (Supporting Information) revealed a single peak for scattering vector *q* near 0.12 Å^−1^, which can be allocated to the lamellar structure of mRNA backbones sandwiched in lipid layers.^[^ ^33^, ^34^ ^]^ The corresponding d‐spacing of M LNPs is calculated to be 5.2 nm. Compared to M LNPs, the peak intensity of M LNP with 5% DOTAP is obviously weaker, which indicates the loss of the well‐ordered lamellar structures after partially replacing structural helper lipid DSPC with DOTAP.

After incubating the LNPs at 42 °C, we observed a clear decrease in the percentage of intact spheres, and reduced to almost 0% for multi‐layer LNPs in both M LNPs and M LNPs with 5% DOTAP samples (Table 1), which potentially indicates those multi‐layer structures have been transformed to “bleb” structures. In contrast, there is a substantial increase in the percentages of “bleb”‐like LNPs, regarded as structural defects of LNPs,^[^ ^35^, ^36^, ^37^ ^]^ which could be induced by lipid composition, buffer excipient and preparation method. Compared to M LNPs after incubation, M LNPs with 5% DOTAP showed a 14.4% higher in percentage of the intact structure, suggesting that the DOTAP replacement stabilized the microstructures of LNPs under external stress from storage conditions.

## 3 Discussion

Access to the two globally‐marketed mRNA‐LNP vaccines manufactured by Pfizer‐BioNTech and Moderna relies heavily on cold‐chain distribution and transport to avoid rapid degradation during non‐frozen storage in solution.^[^ ^9^ ^]^ Identifying the key factors contributing to the instability of mRNA LNPs is the first step in improving their in‐solution storage stability. Several studies have indicated that factors such as mRNA length, capping method and construct design influence mRNA hydrolysis, consequently affecting the degradation of the mRNA‐LNP product.^[^ ^38^, ^39^ ^]^ Recently, a new mechanism involving aldehyde impurities produced by ionizable lipids has been discovered, implicating lipid components in mRNA degradation.^[^ ^19^ ^]^ However, few stability studies have focused on the role of helper lipids, which are present throughout the LNPs, and their impact on the microstructures of mRNA‐LNPs.

In this study, we partially and completely replaced DSPC by three types of charged and neutral lipids to create new mRNA‐LNP formulations for stability study. Introduction of charged lipids into the mRNA‐LNPs could affect their transfection efficacy in different cell lines. M LNPs with 2.5% DOPS significantly altered mRNA transfection efficiency in human healthy epithelial, tumor, and immortalized T cells. This is consistent with the previous research finding that LNPs with DOPS can promote targeting to secondary lymphoid organs and immune cells.^[^ ^40^ ^]^ In contrast, replacement with 5% DOTAP in M LNPs didn't improve the transfection efficacy of M LNPs in HEK293T cells.

To evaluate the stability of mRNA‐LNPs, we characterized size, zeta potential, encapsulation efficiency, integrity of encapsulated mRNA, and mRNA transfection in HEK293T cells upon exposure to 42 °C, RT (22 °C), and 4 °C for up to 12 days, 6 weeks, and 6 months, respectively. We noted that Moderna LNP formulations outperform Pfizer LNP formulations, especially for mRNA transfection performance. This has also been observed in previous studies comparing the two commercial formulations.^[^ ^41^ ^]^ Moreover, we found that replacing half of DSPC by DOTAP can significantly improve mRNA integrity and transfection at 42 °C. The partial replacement with DOTAP increased the in‐cell functional stability of Moderna mRNA‐LNPs at RT, and showed a trend of improvement at 4 °C. The discrepancy of degradation patterns at different temperature conditions indicates that replacement of helper lipids works well for improving mRNA‐LNP stability at storage conditions with elevated temperatures above RT, while at refrigerated conditions with mild stress, other factors such as types of ionizable lipids might play a predominant role.

We investigated the mechanism underlying the storage stability of mRNA‐LNPs using cryo‐EM to elucidate the stability improvement observed when part of DSPC was replaced by DOTAP in the two commercial formulations. Statistical analysis of cryo‐EM images of M LNPs and M LNPs with 5% DOTAP demonstrated the addition of DOTAP can increase the percentages of intact LNPs, while reducing formation of semi‐stable “bleb” structures in the freshly formulated samples. After inducing degradation by incubation at 42 °C, we observed a substantial increase in “bleb” structures, with fewer “blebs” observed in M LNPs with 5% DOTAP. We hypothesize that the partial replacement of DOTAP can stabilize the microstructures of LNPs and improve their resistance to external environmental stress during in‐solution storage.

However, whether “bleb” structures facilitate or deteriorate storage stability of mRNA‐LNPs is still under debate. Cheng et al used citrate buffer with high salt concentrations to formulate mRNA‐LNPs, which displayed mostly “bleb” structures and delayed degradation in presence of serum.^[^ ^29^ ^]^ The condition of their stability test was different from our study, focusing on storage of mRNA‐LNP product in solution for transport and distribution. Another research work from Reinhart et al formulated mRNA‐LNPs using five types of ionizable lipids and compared storage stability for two of them.^[^ ^42^ ^]^ Their results showed in‐cell transfection of C12‐200 LNPs was more stable than SM‐102 LNPs (Moderna) at 2–8 and 25 °C. In our study, we observed more “bleb” structures and less storage stability in Pfizer (ALC‐0315) LNPs as compared to Moderna (SM‐102) LNPs (Figure S14, Supporting Information). Collectively, these findings indicate that chemical structures of ionizable lipids would also play an important role in the storage stability of mRNA‐LNPs, and further mechanistic study is necessary.

## 4 Conclusion

In this work, we replaced part of helper phospholipids DSPC in commercial mRNA‐LNP products by three types of alternative lipids with different charges, and investigated their in‐solution storage stability by incubating them at 42 °C, RT (22 °C), and 4 °C, respectively. Their hydrodynamic size, zeta potential, encapsulation efficiency, mRNA integrity, and transfection in cells were subsequently evaluated. Our results indicate that the mRNA‐LNP degradation primarily occurs through degradation of the mRNA encapsulated in the LNPs, consequently comprising in‐cell performance. From the stability study, we found that replacement with 5% DOTAP could improve the storage stability of mRNA‐LNPs (Moderna) under different temperature conditions. Further mechanistic studies revealed that the DOTAP replacement can decrease the formation of “bleb” structures and provide resistance against “bleb” formation triggered by temperature stress during storage in solution. Also, spacing between mRNA chains was slightly enlarged when DOTAP was introduced. Overall, we identified the important roles of helper lipids in the storage stability of mRNA LNPs, and correlated the stability results with structural analysis, such as cryo‐EM and SAXS. As a proof‐of‐concept study, we proposed a new strategy to improve in‐solution stability of two commercially available mRNA‐LNPs, and correlated structural analysis with their stability performance. Through a detailed study on one of the major lipid components, our findings lay the groundwork for further investigations aimed at identifying the key factor affecting mRNA LNPs stability and reducing reliance on cold chain storage and transport for global distribution.

## 5 Experimental Section

### Materials

Heptadecan‐9‐yl 8‐((2‐hydroxyethyl)(6‐oxo‐6‐(undecyloxy) hexyl) amino) octanoate (SM‐102), 1,2‐Distearoyl‐sn‐glycero‐3‐phosphocholine (DSPC), ((4‐hydroxybutyl)azanediyl)bis(hexane‐6,1‐diyl)bis(2‐hexyldecanoate) (ALC‐0315), 2‐[(polyethylene glycol)‐2000]‐N,N‐ditetradecylacetamide (ALC‐0159) were purchased from SINOPEG, China. 1,2‐Dimyristoyl‐rac‐glycero‐3‐methoxypolyethylene glycol‐2000 (DMG‐PEG2k) was purchased from Cayman Chemical, USA. Cholesterol,1,2‐di‐(9Z‐octadecenoyl)‐sn‐glycero‐3‐phospho‐L‐serine (DOPS), 1,2‐dioleoyl‐3‐trimethylammonium‐propane (DOTAP), and 1,2‐di‐(9Z‐octadecenoyl)‐sn‐glycero‐3‐phosphoethanolamine (DOPE) were purchased from Merck Life Science.

### Cell Culture

HEK293T (RRID: CVCL_0063), MDA‐MB‐231 (RRID: CVCL_0062), and Jurkat (RRID: CVCL_0065) cells were purchased from the American Type Culture Collection (ATCC). HEK293T and MDA‐MB‐231 cells were grown in Dulbecco's modified Eagle medium (ThermoFisher) supplemented with 10% fetal bovine serum (ThermoFisher) and 1 % penicillin‐streptomycin (ThermoFisher). Jurkat cells were grown in Roswell Park Memorial Institute 1640 medium (ThermoFisher) with the same supplements. All the cells were cultured at 37 °C and 5% CO_2_ with saturated humidity, and tested to be free of mycoplasma contamination (Lonza) prior to cell experiments.

### Production of mRNA

A single batch of mRNA encoding enhanced green fluorescent protein (mRNA‐eGFP) was manufactured by BASE mRNA facility, University of Queensland, Australia, according to their standard procedures of production. In brief, gBlock template for GFP was used to generate PCR product, followed by in vitro transcription to produce mRNA. Cap1 analog (TriLink) and N1‐methyl‐pseudouridine modification were also incorporated. An mRNA purity of 97.30% was obtained by ion‐pair PATfix high‐performance liquid chromatography (BIA Separations), and the size of mRNA was confirmed by TapeStation (Agilent). In addition, endotoxin levels of the purified mRNA were measured with an eEndosafe endotoxin testing kit (Charles River Laboratory), and an endotoxin level of <0.331 EU/mg was recorded.

### Lipid Nanoparticle Formulation and Characterization

Lipid nanoparticles carrying mRNA were formulated as previously described.^[^ ^43^ ^]^ For mRNA‐LNPs (Moderna), SM‐102, DSPC, cholesterol, DMG‐PEG2000 and other alternative lipids (DOPS, DOTAP or DOPE) were dissolved in ethanol at a molar ratio of 50:10‐*x*:38.5:1.5:*x*, where *x* = 0, 2.5, 5, or 10 (total lipid concentration: 2.68 mm). For mRNA LNPs (Pfizer‐BioNTech), ALC‐0315, DSPC, cholesterol, ALC‐0159 and the alternative lipids were kept at a molar ratio of 46.3:(9.4‐x):42.7:1.6:x, where *x* = 0, 2.35, 4.7 or 9.4 (total lipid concentration: 2.9 mm). LNPs were prepared by mixing mRNA in 100 mm citrate buffer (pH 4.0) and the ethanol solution of lipids at a volume ratio of 3:1, while N/P ratio was kept at 5.67. DOPS has low solubility in ethanol, so it was dissolved in chloroform with a concentration of 10 mm before adding to lipid mixture in ethanol. All LNPs were transferred with a dialysis membrane tubing with a molecular weight cut‐off (MWCO) of 10 kDa (ThermoFisher), then dialyzed against 100 mm phosphate buffer saline (PBS, pH 7.4) at 4 °C for 24 h.

To provide quality control for the freshly formulated LNPs, hydrodynamic size and zeta potential of the mRNA LNPs in the PBS were characterized by ZetaSizer Ultra (Malvern Panalytical). Encapsulation efficiency was evaluated using Quant‐it RiboGreen assay kit (ThermoFisher) according to the manufacturer's protocol.

### Stability Study

The as‐prepared mRNA‐LNPs solutions (mRNA concentration: 20 µg mL^−1^) were stored in RNase‐free vials, then incubated at different temperature conditions. Samples were collected at specified time intervals, i.e., every 4 days at 42 °C, every 2 weeks at RT, and every 1 month at 4 °C. Stability was evaluated by characterizing size, zeta potential, encapsulation efficiency, mRNA integrity, and transfection efficiency on HEK293T cells of the collected samples. Three independent experiments including preparation of mRNA‐LNPs, storage and analysis were performed for each temperature condition.

### Evaluation of Intact mRNA by Automated Electrophoresis

The collected mRNA LNPs samples were lysed by adding 1% Triton X‐100 (Sigma) and incubating on ice for 30 min. Total RNA (>17 nt) was extracted by RNA clean & concentrator kit (Zymo Research) and eluted by 5 µL RNase‐free water according the protocol provided by manufacturer. NanoDrop (Implen) was used to measure concentration and quality index of the extracted RNA. Then, 25 ng of RNA was loaded to TapeStation system (Agilent) for automated electrophoresis. Percentages of intact mRNA = (area under mRNA peak size ± 20%) / (Total RNA area exclude lower maker) × 100%.

### In Vitro Transfection on HEK293T Cells

HEK293T cells were seeded on a 24‐well plate at a density of 1 × 10^5^ per well. After 24 h, mRNA‐LNPs containing 250 ng RNA were added to each well and incubated for another 24 h. Then, the cells were treated with 0.5% Trypsin (ThermoFisher) and analysed by a flow cytometer (BD FACS Canto II) to evaluate their mean fluorescence intensity of green fluorescence.

### Cell Viability Assay

HEK293T cells were seeded into 96‐well plates at a density of 1 × 10⁴ cells per well. To assess the potential cytotoxicity of DOTAP in LNP formulation, M LNPs, M LNPs with 5% DOTAP, P LNPs, and P LNPs with 4.7% DOTAP were freshly prepared and added to the cells at final mRNA concentrations of 7.8125, 15.625, 31.25, 62.5, and 125 ng mL^−1^. Each group contains five replicates, and non‐treated cells were set as control group. After 24 h incubation, the cell culture medium was replaced by fresh cell medium containing cell proliferation reagent WST‐1 (Roche). After 2 h, absorbance at 450 nm was measured using a microplate reader Cell viability was calculated by normalization with control group.

### Cryogenic Electron Microscopy

To prepare samples for cryogenic electron microscopy (cryo‐EM), an ultra‐centrifugal filter unit (Millipore) was used to concentrate mRNA LNPs to 10 times their original concentration (final concentration was ≈5 × 10^10^ mL^−1^ as measured by nanoparticle tracking analysis). Grids were glow discharged at 20 mA for 30 s prior to use (GloQube Plus Glow Discharge System, Quorum Technologies Ltd). LNPs were vitrified by applying 3 µL samples to lacey carbon 300 Cu mesh grids, approximate grid hole size 63 microns (PELCO TEM). A Vitrobot mark IV (Thermo Scientific) was used for vitrification, with the chamber maintained at 100% humidity and 12 °C; following application, samples were equilibrated for 30 s, then blotted for 3–4 s prior to plunge cooling in liquid ethane. Samples were screened and imaged on a Glacios cryo‐EM (Thermo Scientific) operating at an accelerating voltage of 200 kV, equipped with a zero‐loss Selectris energy filter with a slit width of 10 eV and Falcon IV camera operating in counting mode. Images were acquired at 79000 × nominal magnification, 5 second exposure time, dose rate ≈4 e Å^−2^ s^−1^ and defocus of −1.8 to −2 microns with a total dose of ≈15–20 e Å^−^2

### Small Angle X‐Ray Scattering (SAXS)

Synchrotron SAXS experiments were performed at the SAXS/WAXS beamline at the Australian Synchrotron, ANSTO, using a wavelength of *k* = 1.128 Å (11.0 keV) and a camera length of 1.6 or 2.5 m as previously described.^[^ ^31^ ^]^ The empty LNPs and LNPs with DOTAP (i.e., without mRNA, lipid concentration was ≈5 mg mL^−1^) were freshly prepared and dispersed in 50 mm citrate buffers (pH 3, 4 and 5.5) and 50 mm PBS (pH 7.4). The mRNA LNPs and mRNA LNPs with DOTAP (lipid concentration was ≈5 mg mL^−1^) were also prepared and dispersed in 50 mm PBS (pH 7.4). Then all the samples were loaded into a UV‐clear 96 well plate (Greiner Bio‐One, Germany) and screened with a 2 s exposure time. A Dectris‐Pilatus 1 m detector was used to record the 2D X‐ray diffraction patterns, and the images were integrated into 1D graphs plotting intensity versus the scattering vector, *q*, using the ScatterBrain (V 1.230) software. The scattering vector, *q*, is defined by *q *= 4πsinθ/λ where θ is the scattering angle and λ is the wavelength. According to Bragg's law, the spacing *d* was calculated as *d *= 2π/*q*.

### Statistical Analysis

Data analysis in this study was performed using GraphPad Prism version 10 software. Data was presented as mean ± standard error of the mean (SEM). Statistical significance in mean values between two groups was determined by unpaired t‐test. For comparison that involves more than two groups, one‐way or two‐way analysis of variance (ANOVA) was used. *p* <0.05 or ‐log (p‐value) > 1.301 indicated statistical significance.

## Conflict of Interest

The authors declare no conflict of interest.

## Author Contributions

R.C., P.E. and C.X.Z. conceived the project and designed the experiments. X.W. supported nanoparticle preparation and characterization. R.C., L.X. and N.Z. performed the experiments. F.W. prepared cryo‐EM samples. F.W. and R.C. imaged nanoparticles by cryo‐EM. H.Y. and J.Z. performed SAXS and analysis. R.C. performed data analysis, prepared figures and drafted the manuscript. H.Y., J.Z., P.E. L.X. and C.X.Z. revised the manuscript. All authors have contributed to editing the manuscript and given approval to the final version of the manuscript.

## Supporting information



Supporting Information

## Data Availability

The data that support the findings of this study are available from the corresponding author upon reasonable request.
