# Supplementary material for: Replacing Helper Lipids With Cationic Lipids Enhances mRNA Lipid Nanoparticles Stability in Solution
Source: Adv Sci (Weinh). 2025 Oct 30;13(3):e11637. doi: 10.1002/advs.202511637 (PMC12806310; doi:10.1002/advs.202511637)
Supplement: Supplementary file 1 — Supporting Information [file ADVS-13-e11637-s001.docx]

Supporting information for

**Replacing helper lipids with cationic lipids enhances mRNA lipid nanoparticles stability in solution**

AUTHOR NAMES

Rui Chen^†^, Letao Xu^†^, Nan Zhang^†^, Haitao Yu^‡^, Xing Wang^†^, Jiali Zhai^‡^, Fiona Whelan^§^, Phillip Elliott^‖^, Chun-Xia Zhao^†*^

ADDRESS

†School of Chemical Engineering, Faculty of Sciences, Engineering and Technology, The University of Adelaide, Adelaide, South Australia 5005, Australia

‡School of Science, STEM College, RMIT University, Melbourne, Victoria 3000, Australia

§Department of Molecular and Biomedical Science, and Adelaide Microscopy, University of Adelaide, Adelaide, South Australia 5005, Australia.

^‖^BioCina Pty Ltd, Thebarton, South Australia 5031, Australia

Address correspondence to:

Chun-Xia Zhao, Prof.

The University of Adelaide

Email: [chunxia.zhao@adelaide.edu.au](mailto:chunxia.zhao@adelaide.edu.au)


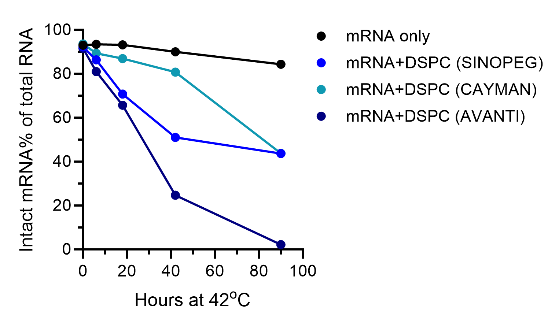

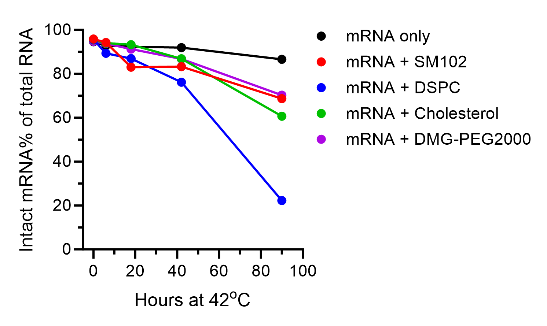


**A**

**B**

**Figure S1.** Degradation of mRNA incubated with lipid components of mRNA LNPs. (A) Percentages of intact mRNA after incubating 5 ug mRNA-eGFP with lipid components: SM102 (90 nmol), DSPC (18 nmol), cholesterol (69.3 nmol) and DMG-PEG2000 (2.7 nmol) at 42^°^C characterized by electrophoresis. (B) Percentages of intact mRNA after incubation with DSPC (18 nmol) from three suppliers including SINOPEG, CAYMAN and AVANTI at 42^°^C.


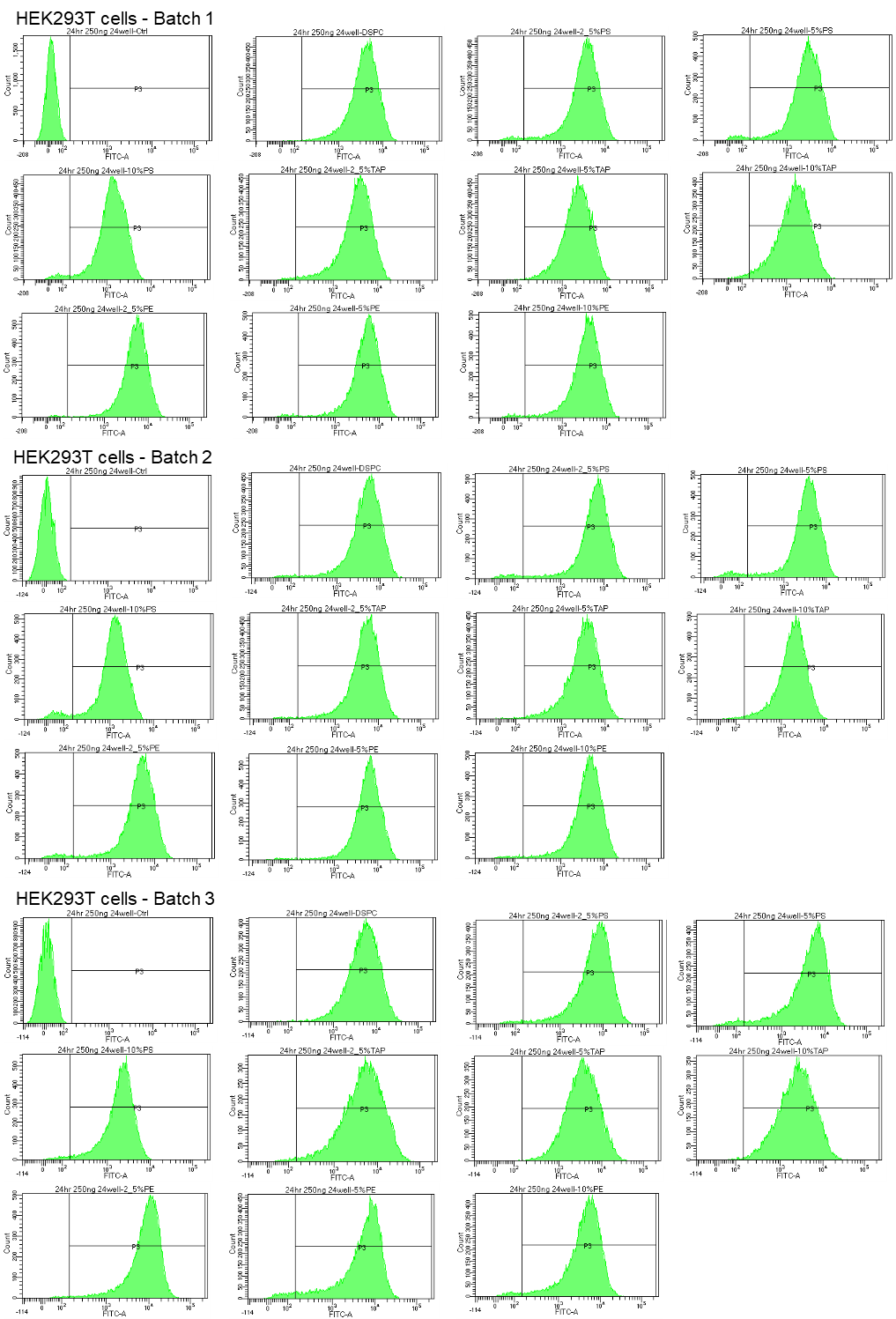
**Figure S2.** Flow cytometry analysis for HEK293T cells treated with M LNPs and M LNPs with helper lipids replaced by DOPS, DOTAP and DOPE.
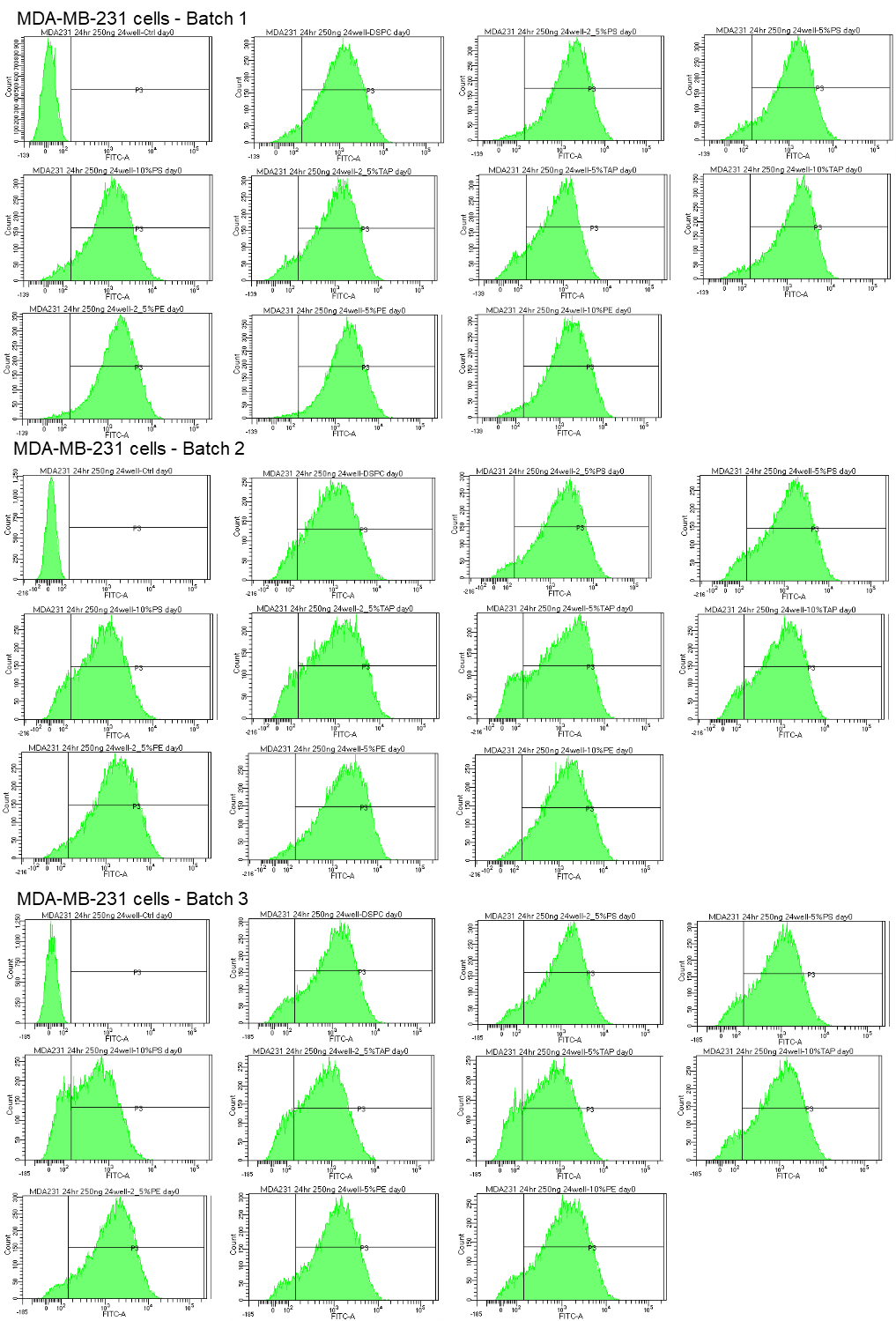
**Figure S3.** Flow cytometry analysis for MDA-MB-231 cells treated with M LNPs and M LNPs with helper lipids replaced by DOPS, DOTAP and DOPE.
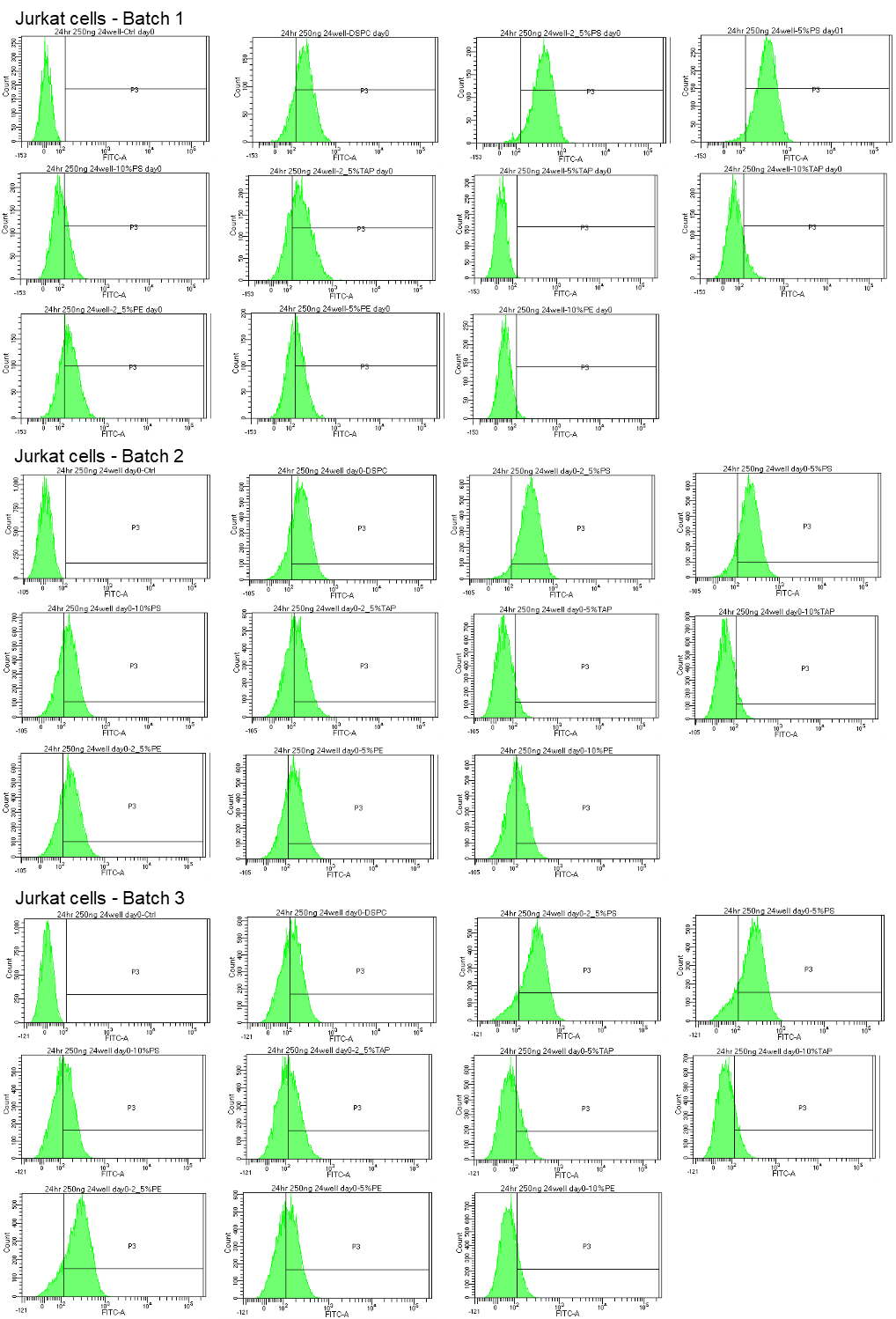
**Figure S4.** Flow cytometry analysis for Jurkat cells treated with M LNPs and M LNPs with helper lipids replaced by DOPS, DOTAP and DOPE.

**Figure S5.** Cell viability of HEK293T cells treated with (A) M LNPs, M LNPs with 5% DOTAP, (B) P LNPs and P LNPs with 4.7% DOTAP.

**A**

**B**

**Figure S6.** Stability study of M LNPs, P LNPs and their formulation with DOTAP replacement at room temperature (RT) and 4^°^C. LNPs and conditions include (A) M LNPs at RT, (B) P LNPs at RT, (C) M LNPs at 4^°^C, and (D) P LNPs at 4^°^C. Evaluations from left to right hand side: hydrodynamic size, zeta potential, encapsulation efficiency and RNA electrophoresis of the encapsulated mRNA.

**A**

**B**

**D**

**C**

**
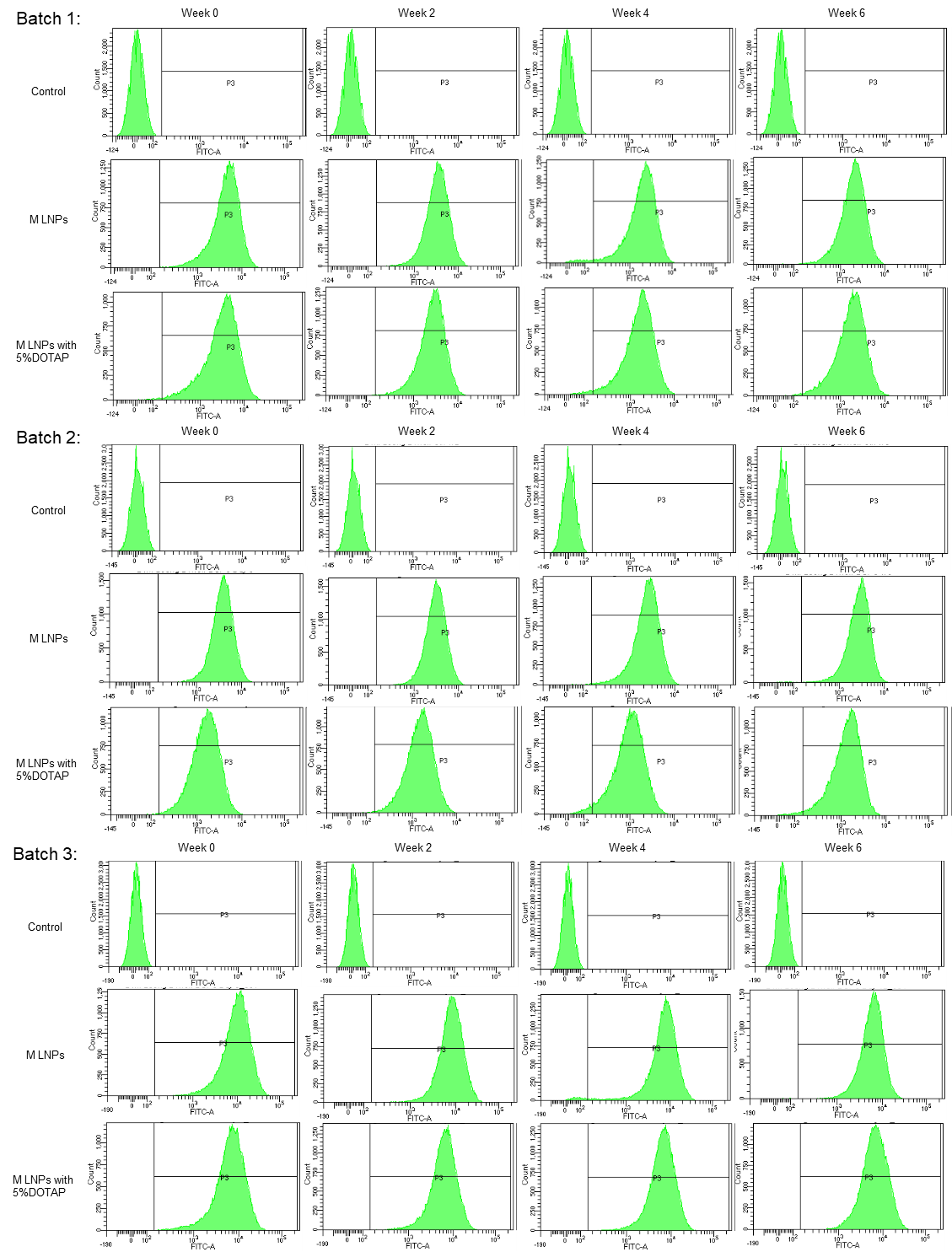
Figure S7.** Flow cytometry analysis of HEK293T cells for stability study of M LNPs and M LNPs with 5% DOTAP stored at RT.

**
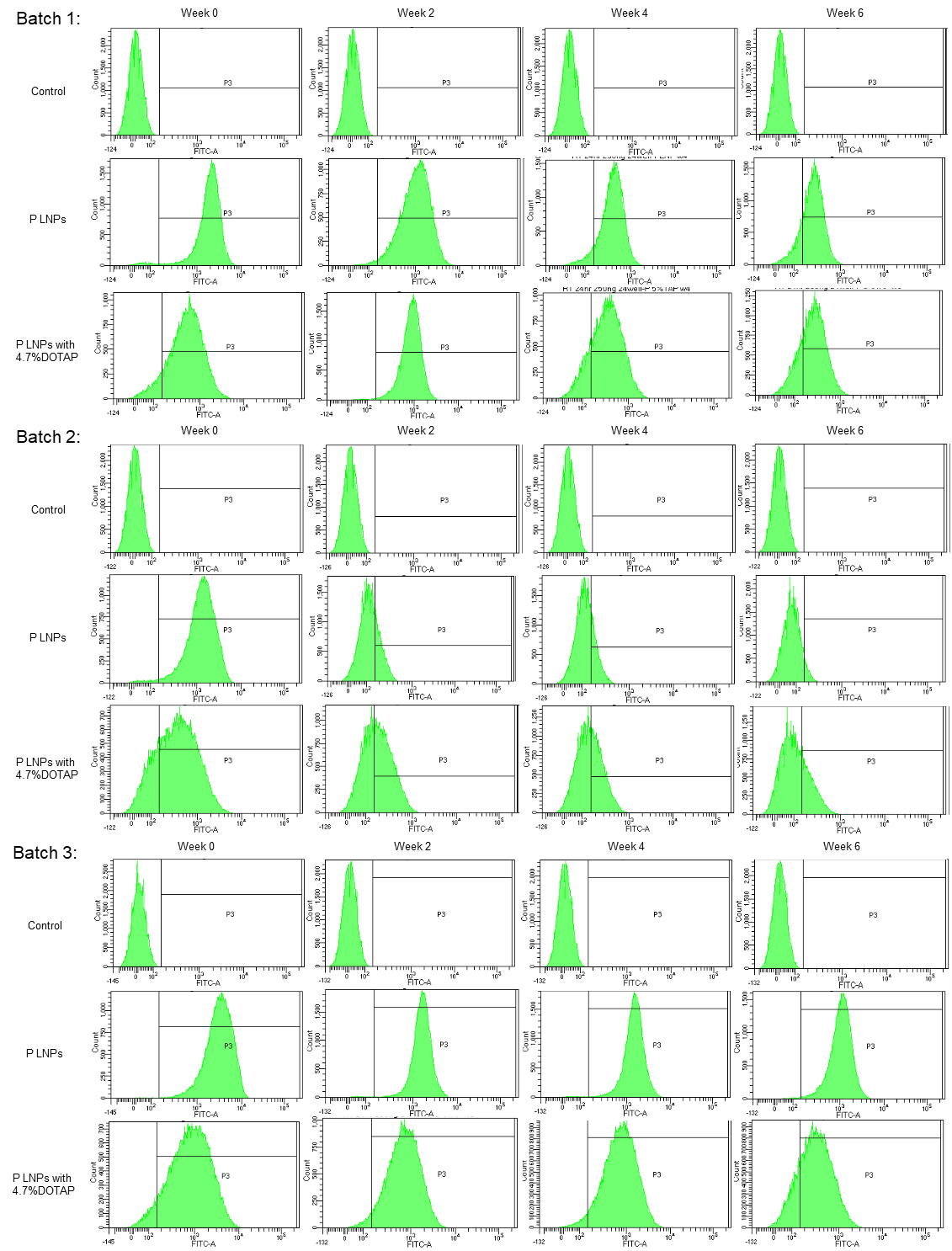
Figure S8.** Flow cytometry analysis of HEK293T cells for stability study of P LNPs and P LNPs with 4.7% DOTAP stored at RT.

**
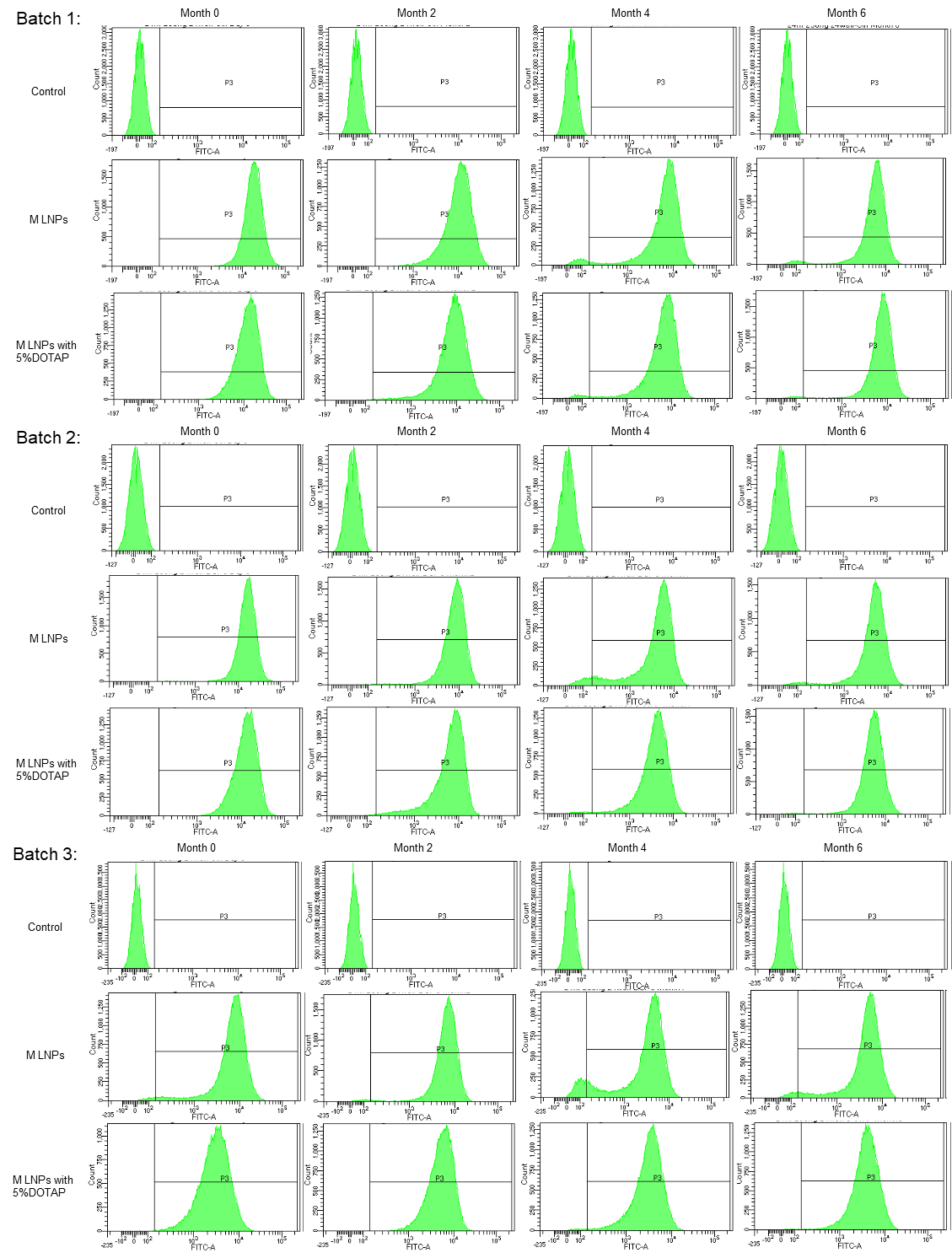
Figure S9.** Flow cytometry analysis of HEK293T cells for stability study of M LNPs and M LNPs with 5% DOTAP stored at 4^°^C.

**
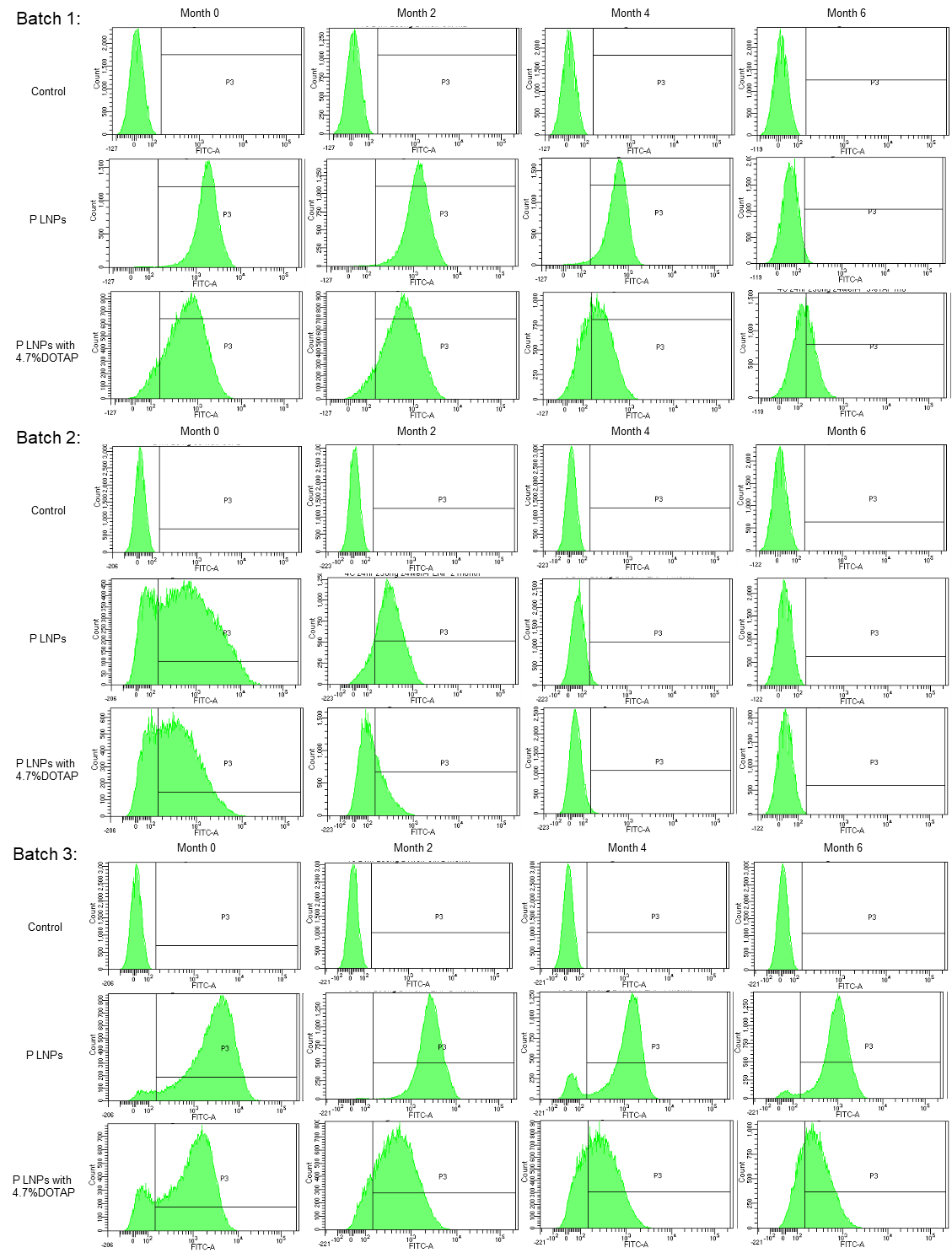
****Figure S10.** Flow cytometry analysis of HEK293T cells for stability study of P LNPs and P LNPs with 4.7% DOTAP stored at 4^°^C.


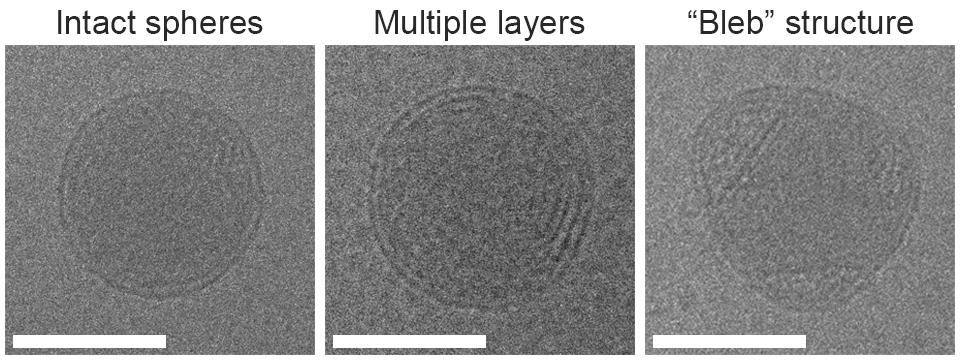


**Figure S11.** Cryo-EM images for three typical microstructures identified in mRNA LNPs. Scale bar = 50 nm.


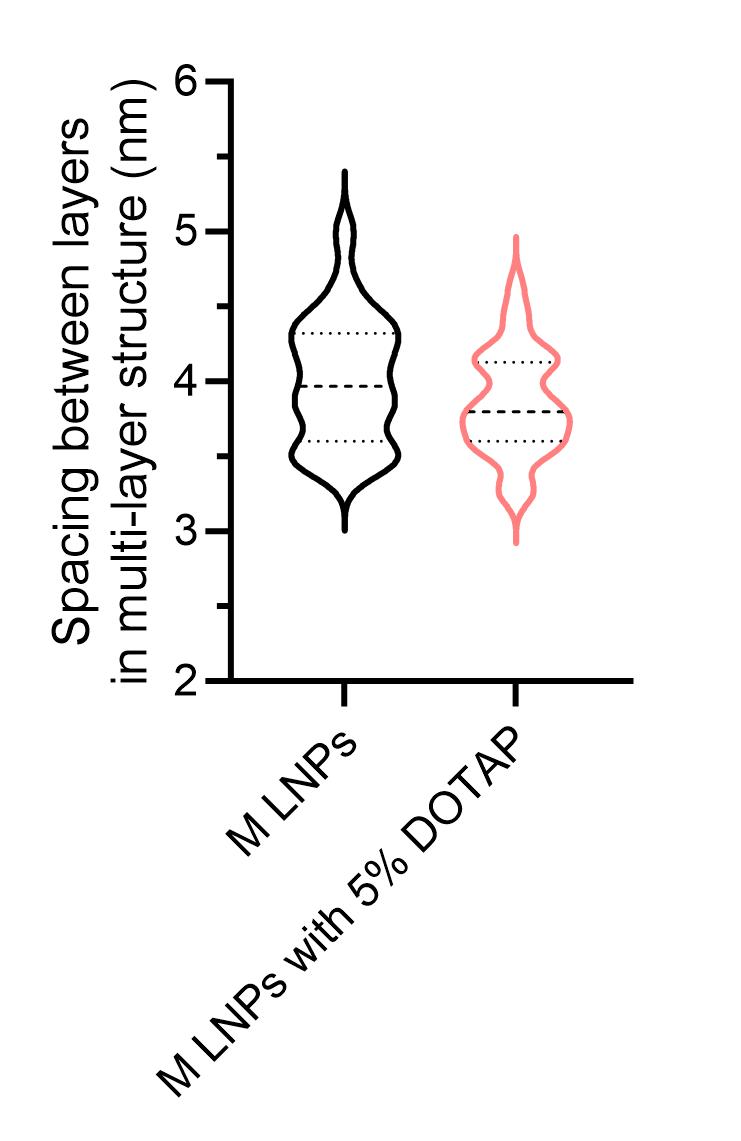


**Figure S12.** Layer spacing of multiple-layer structure in cryo-EM images of M LNPs and M LNPs with 5% DOTAP.


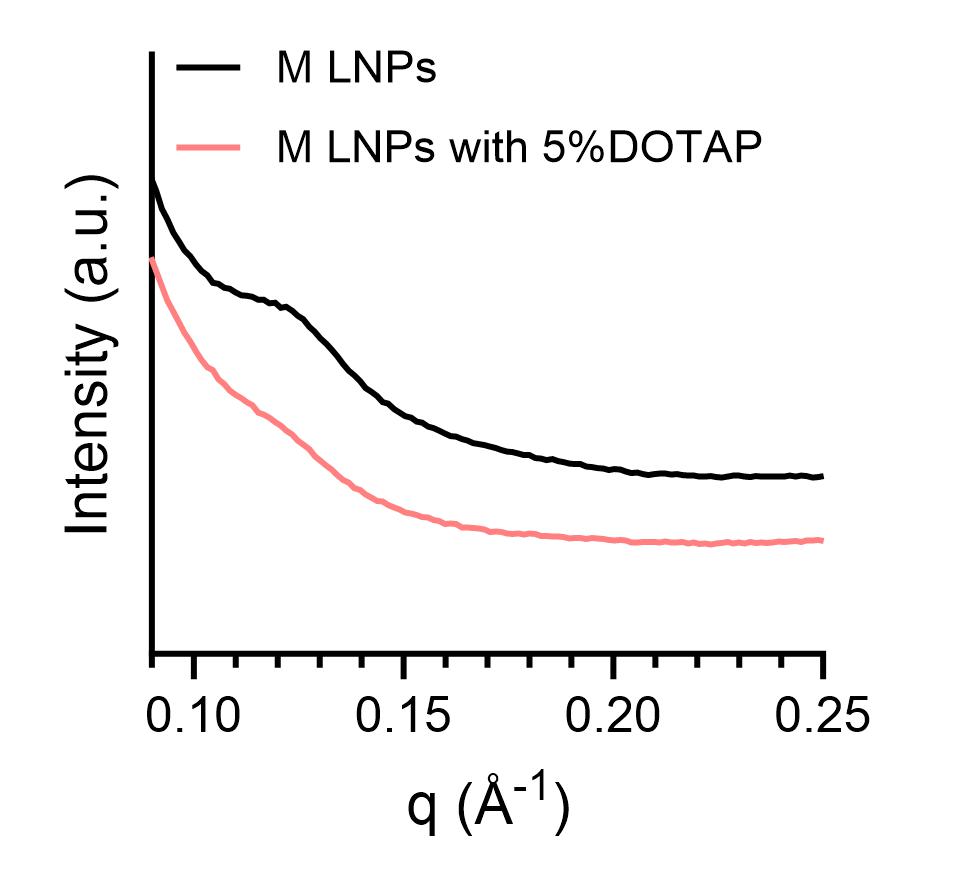
**Figure S13.** Synchrotron SAXS patterns for M LNPs and M LNPs with 5% DOTAP in PBS buffer solution at 25 ^°^C.


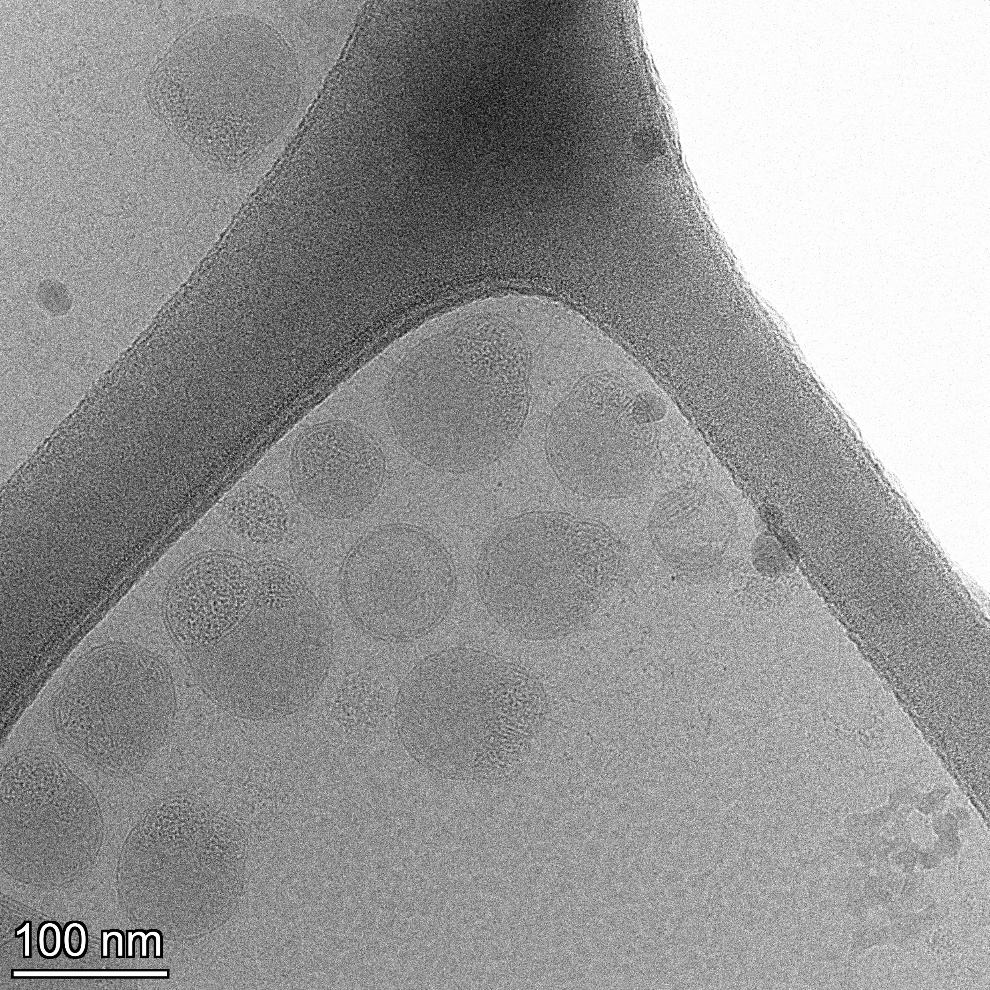

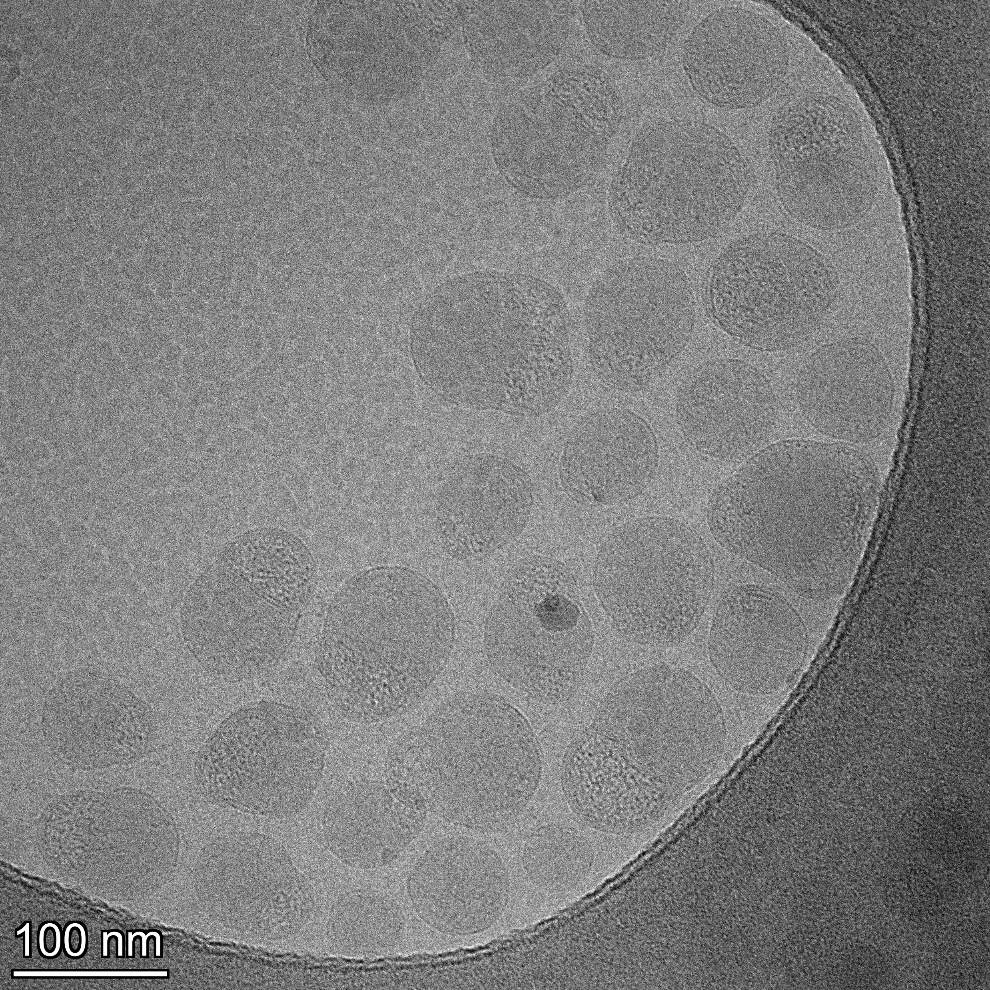

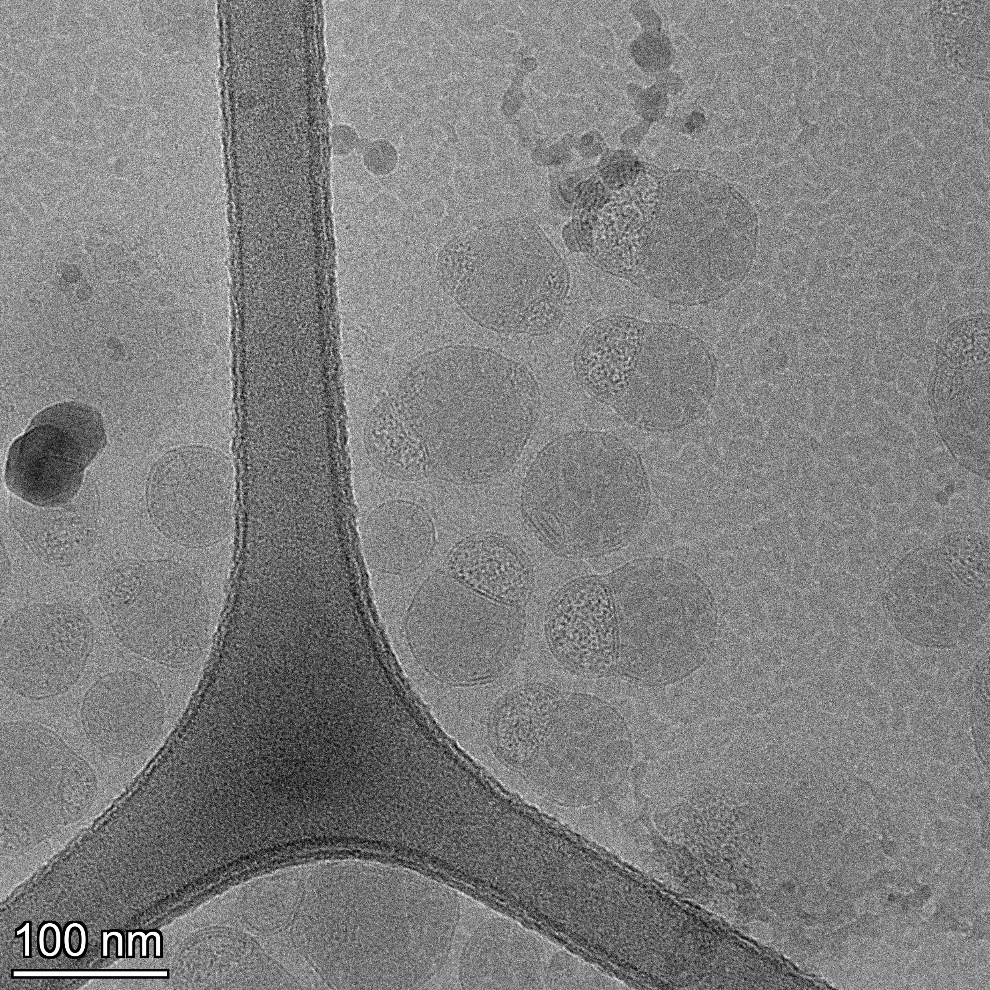

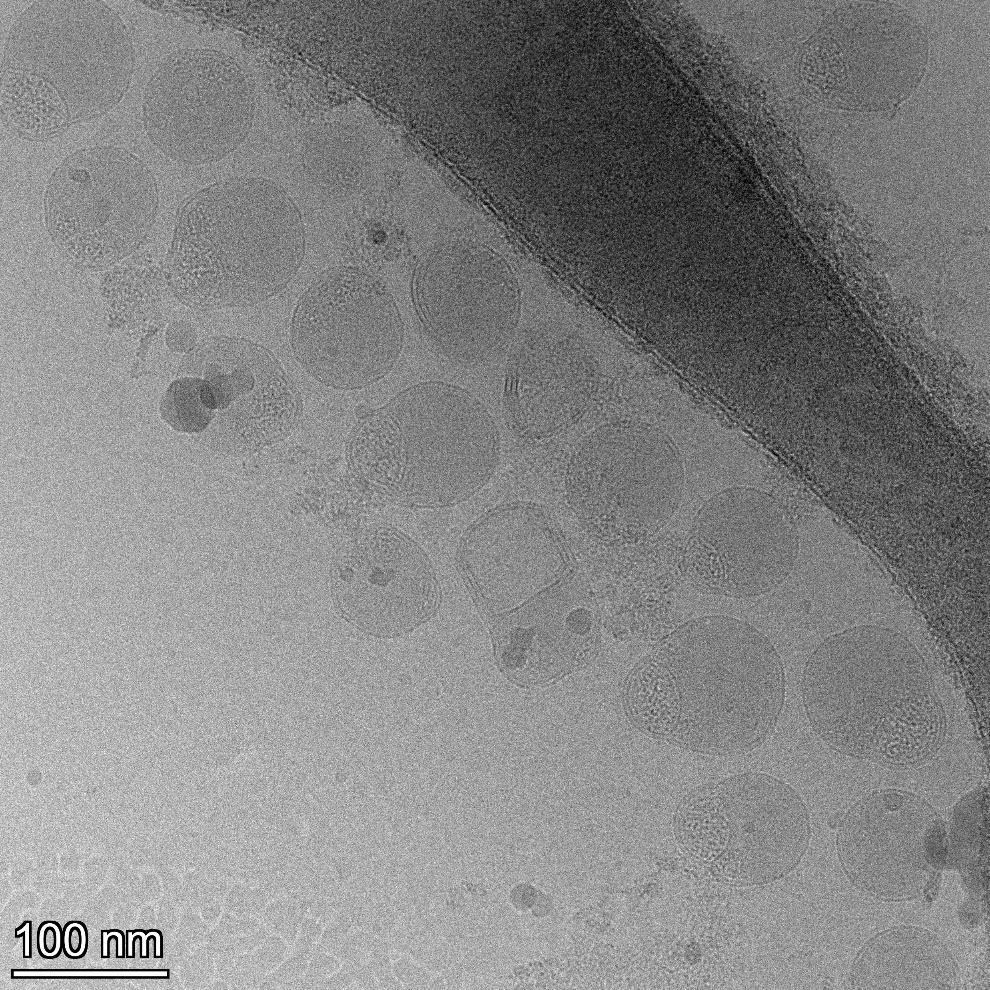


**A**

**B**

**Figure S14.** Cryo-EM images of (A) P LNPs and (B) P LNPs with 4.7% DOTAP.
